# Fumonisin B_2_ Induces Mitochondrial Stress and Mitophagy in Human Embryonic Kidney (Hek293) Cells—A Preliminary Study

**DOI:** 10.3390/toxins14030171

**Published:** 2022-02-25

**Authors:** Jivanka Mohan, Naeem Sheik Abdul, Savania Nagiah, Terisha Ghazi, Anil A. Chuturgoon

**Affiliations:** 1 Discipline of Medical Biochemistry, School of Laboratory Medicine and Medical Sciences, University of KwaZulu-Natal, Durban 4041, South Africa; mxtjivee@gmail.com (J.M.); naeemsheik11@gmail.com (N.S.A.); Nagiah.savania@gmail.com (S.N.); terishaghazi@gmail.com (T.G.); 2 Applied Microbial and Health Biotechnology, Cape Peninsula University of Technology, Cape Town 7535, South Africa; 3 Medical Programme, Department of Human Biology, Faculty of Health Sciences, Nelson Mandela University Missionvale, Bethelsdorp, Port Elizabeth 6059, South Africa

**Keywords:** fumonisin B_2_, mitophagy, mitochondrial stress, human kidney cells, miR-27b

## Abstract

Ubiquitous soil fungi parasitise agricultural commodities and produce mycotoxins. Fumonisin B_2_ (FB_2_), the structural analogue of the commonly studied Fumonisin B_1_ (FB_1_), is a neglected mycotoxin produced by several *Fusarium* species. Mycotoxins are known for inducing toxicity via mitochondrial stress alluding to mitochondrial degradation (mitophagy). These processes involve inter-related pathways that are regulated by proteins related to SIRT3 and Nrf2. This study aimed to investigate mitochondrial stress responses in human kidney (Hek293) cells exposed to FB_2_ for 24 h. Cell viability was assessed via the methylthiazol tetrazolium (MTT) assay, and the half-maximal inhibitory concentration (IC_50_ = 317.4 µmol/L) was estimated using statistical software. Reactive oxygen species (ROS; H_2_DCFDA), mitochondrial membrane depolarisation (JC1-mitoscreen) and adenosine triphosphate (ATP; luminometry) levels were evaluated to assess mitochondrial integrity. The relative expression of mitochondrial stress response proteins (SIRT3, pNrf2, LONP1, PINK1, p62 and HSP60) was determined by Western blot. Transcript levels of *SIRT3*, *PINK1* and miR-27b were assessed using quantitative PCR (qPCR). FB_2_ reduced ATP production (*p* = 0.0040), increased mitochondrial stress marker HSP60 (*p* = 0.0140) and suppressed upregulation of mitochondrial stress response proteins SIRT3 (*p* = 0.0026) and LONP1 (*p* = 0.5934). FB_2_ promoted mitophagy via upregulation of pNrf2 (*p* = 0.0008), PINK1 (*p* = 0.0014) and p62 (*p* < 0.0001) protein expression. FB_2_ also suppressed miR-27b expression (*p* < 0.0001), further promoting the occurrence of mitophagy. Overall, the findings suggest that FB_2_ increases mitochondrial stress and promotes mitophagy in Hek293 cells.

## 1. Introduction

Favourable weather conditions cause ubiquitous soil fungi to parasitise agricultural commodities and produce mycotoxins [1]. Ingestion of mycotoxin-contaminated food can induce adverse effects on the health of the consumer. Mycotoxin consumption is associated with acute and chronic toxicity in both humans and animals [1,2]. 

Fumonisin B_2_ (FB_2_) is the structural analogue of the popularly studied fumonisin B_1_ (FB_1_). Fumonisins are highly prevalent toxins produced mainly by the *Fusarium* species, a common contaminant of maize [3]. These mycotoxins have been classified by the International Agency for Research on Cancer as class 2B carcinogens, indicating potential toxicity to humans [4]. FB_1_ has been implicated in several pathologies in humans [5,6,7,8]. The toxin exerts its adverse effects in humans via numerous pathways, including induction of apoptosis, oxidative stress, mitochondrial dysfunction, and epigenetic modifications [9,10,11,12].

Previous statistics indicated a provisional maximum tolerable intake of 2 µg/kg for FB_1_ and FB_2._ However, a more recent study suggests a tolerable daily intake of 1.0 μg/kg following analysis in mice [13]. The World Health Organization has reported that developed countries have low exposure to fumonisins. However, exposure often exceeds the provisional maximum tolerable intake of 1–2 µg/kg in developing countries such as South Africa, Nigeria, Malawi and China, with daily exposure exceeding 15 µg/kg body weight [14].

Despite the high prevalence of FB_2_, there is a dearth of toxicity studies on FB_2_ compared to FB_1_. FB_2_ has a high polarity that allows it to be rapidly excreted via the kidneys as it is highly water-soluble. This causes the kidney to be susceptible to FB_2_-induced toxicity [15]. Previous studies using equine tissue (brain, liver and kidney) have demonstrated the ability of FB_2_ to inhibit de novo sphingolipid biosynthesis [16]; however, no data currently exist on its physiological and biochemical effects in humans.

Furthermore, limited evidence is available on fumonisin exposure and its effects on mitochondria. Mitochondrial function is imperative to overall cell health [17], and loss of function results in many adverse effects, including decreased ATP concentrations, increased oxidative stress and cell death [18]. Upon exposure to toxins, mitochondria activate antioxidant and mitochondrial stress responses [19]. Proteins such as Heat Shock protein 60 (HSP60), Sirtuin 3 (SIRT3) and Lon protease (LONP1) are activated to maintain mitochondrial function. Upregulation of these proteins can directly ameliorate mitochondrial stress or promote increases in other stress response proteins [20,21,22].

Failure to reduce mitochondrial stress results in mitophagy. Mitophagy allows maintenance of cellular homeostasis by degradation of damaged mitochondria. [23]. PTEN-induced putative kinase 1 (PINK1) and ubiquitin-binding adaptor p62 (p62) are proteins necessary for the progression of mitophagy, and their expression is influenced by several promoters and inhibitors [24,25]. 

Nuclear factor (erythroid-derived 2)-like 2 (Nrf2) is a transcription factor commonly associated with an antioxidant response; however, recently, its role in mitophagy promotion has been highlighted. Nrf2 has been shown to transcriptionally regulate *PINK1* and p62 during oxidative stress conditions through the activation of an antioxidant response element sequence in their promoter region [26,27]. Additionally, p62 has been shown to promote Nrf2 activation via inactivation of its cytoplasmic sequestrant, Kelch like-ECH-associated protein, creating a positive feedback axis [28]. The Nrf2-PINK1-p62 axis promotes cell survival [26,27].

Conversely, small non-coding RNAs, namely micro-RNAs (miRNAs), have been implicated in the negative regulation of mitophagy. MiR-27b expression leads to inhibition of PINK1 expression at a translational level by directly binding to the 3′-untranslated region (3′-UTR) of its messenger RNA, thus post-transcriptionally regulating mitophagy [29].

The mechanism of FB_2_-induced mitochondrial stress responses in kidney cells remains unclear. Furthermore, since fumonisins are implicated in mitochondrial toxicity, it would be beneficial to understand the potential of FB_2_ in inducing/promoting mitophagy. This study aimed to determine the effects of FB_2_ on the mitochondrial stress responses and mitophagy in human kidney (Hek293) cells by determining mitochondrial output and mitochondrial maintenance. 

## 2. Results

### 2.1. Cytotoxicity of FB_2_ in Hek293 Cells

**Figure 1:**
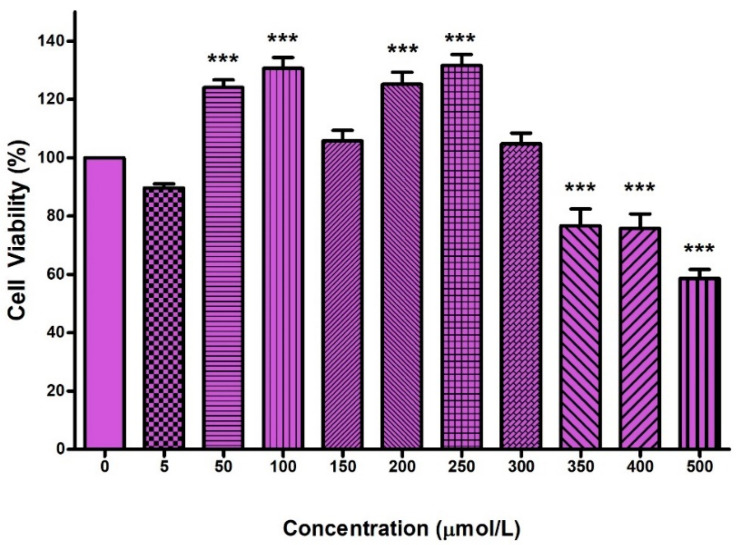
FB2 cytotoxicity in Hek293 cell after 24 h of treatment. *** *p* < 0.0001 relative to control (0 µM).

Cytotoxicity was determined using serially diluted concentrations of FB_2_ (0–500 µmol/L) in Hek293 cells over 24 h (Figure 1). The dose–response curve analysis (non-linear regression) estimated that 317.4 µmol/L of FB_2_ induced 50% cell death in Hek293 cells (IC_50_).

### 2.2. FB_2_ Increased ROS Production and Mitochondrial Membrane Depolarisation in Hek293 Cells

**Figure 2:**
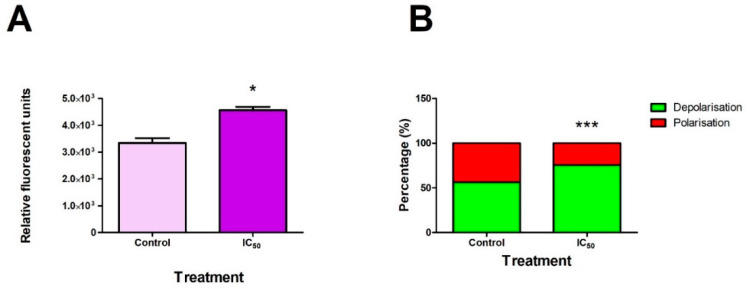
FB_2_ increased ROS production and mitochondrial membrane depolarisation in Hek293 cells. ROS production was significantly increased by FB_2_ ((**A**); * *p* < 0.05) with a corresponding increase in mitochondrial membrane depolarisation ((**B**); *** *p* < 0.0001).

Compromised mitochondrial function may result in exacerbated ROS production that further induces mitotoxicity. ROS production increased significantly (*p* = 0.0156) following exposure with FB_2_ (Figure 2A). Mitochondrial functionality can be observed by quantifying mitochondrial membrane depolarisation. FB2 caused a significant increase in mitochondrial membrane depolarisation in Hek293 cells (*p* < 0.0001) (Figure 2B).

### 2.3. FB_2_ Induces Mitochondrial Stress in Hek293 Cells

**Figure 3:**
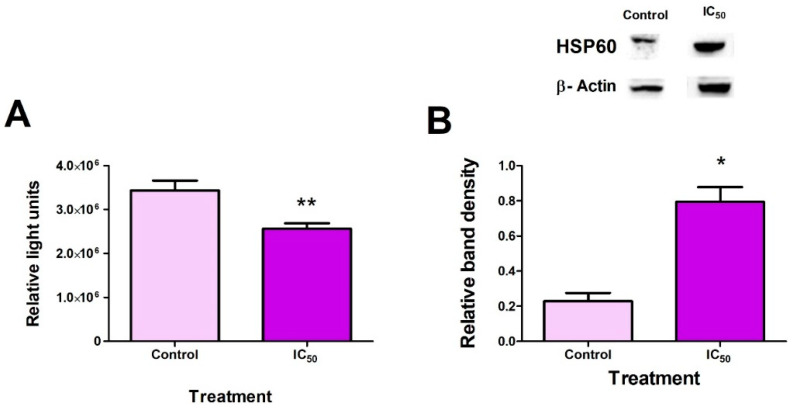
FB_2_-induced mitochondrial stress. FB_2_ significantly decreased ATP levels in Hek293 cells ((**A**); ** *p* < 0.005). HSP60 protein expression increased substantially in Hek293 cells ((**B**); * *p* < 0.05).

We next determined the effects of FB_2_ on mitochondrial stress. To determine the effect of FB_2_ on mitochondrial stress, ATP quantification was carried out (mitochondrial output and functionality), and HSP60 protein expression (a marker for mitochondrial stress) was assessed via Western blots [22]. A significant decrease (*p* = 0.0040) in ATP production compared to the control was observed (Figure 3A). Furthermore, FB_2_ induced a considerable increase (*p* = 0.0140) in HSP60 protein expression, suggesting increased mitochondrial stress (Figure 3B). 

### 2.4. FB_2_ Suppresses Mitochondrial Stress Responses in Hek293 Cells

**Figure 4:**
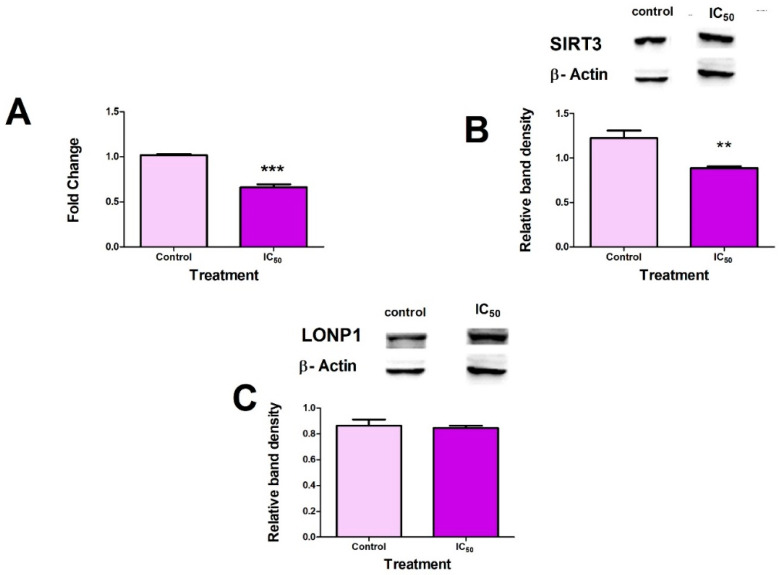
FB_2_ suppressed mitochondrial stress responses in Hek293 cells. FB2 inhibited SIRT3 gene expression in Hek293 cells ((**A**); *** *p* < 0.0001). SIRT3 protein expression was significantly downregulated by FB_2_ in Hek293 cells ((**B**); ** *p* < 0.005). LONP1 protein expression showed no significant changes (**C**).

To confirm the induction of mitochondrial stress, protein (Western blots) and mRNA (qPCR) levels of SIRT3 were analysed. Additionally, protein expression of LONP1 was measured. FB_2_ induced a significant decrease in *SIRT3* mRNA expression (*p* < 0.0001) (Figure 4A) with coinciding decreases in SIRT3 protein expression (*p* = 0.0026) (Figure 4B). No significant changes were observed for LONP1 protein expression (*p* = 0.5934) (Figure 4C).

### 2.5. FB_2_ Activates Nrf2 in Hek293 Cells

**Figure 5:**
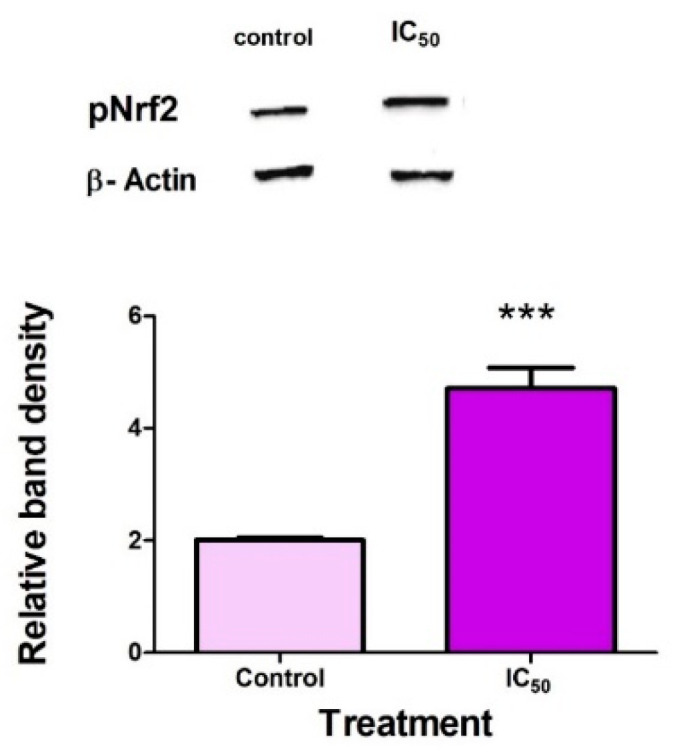
FB_2_ promoted mitophagy. FB_2_ significantly upregulated pNrf2 expression in Hek293 cells (*** p < 0.0001).

Phosphorylated Nrf2 (Ser40) (pNrf2) is the stable and activated form of Nrf2 that has dissociated from KEAP1, allowing it to translocate to the nucleus and transcribe for proteins [30,31]. Following FB_2_ exposure, the expression of pNrf2 was significantly increased (*p* = 0.0008) (Figure 5). 

### 2.6. FB_2_ induced Mitophagy in Hek293 Cells

**Figure 6:**
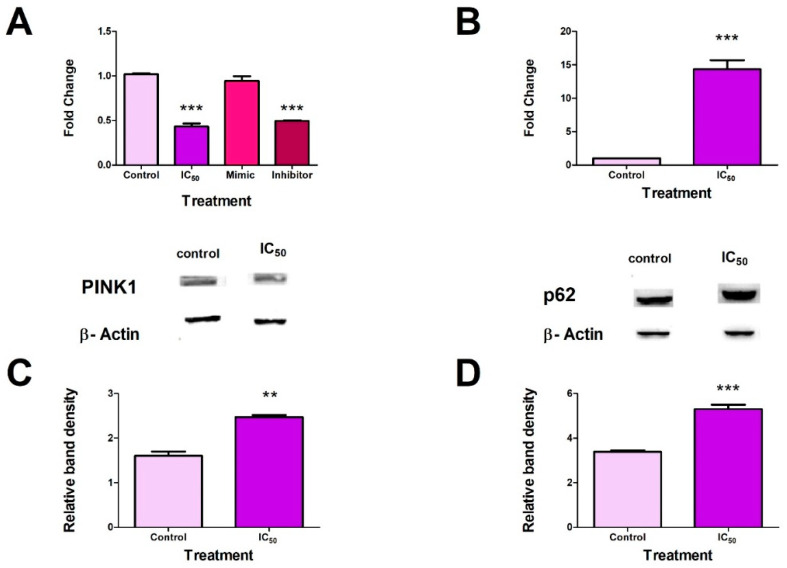
FB_2_ promoted mitophagy in Hek293 cells. FB_2_ suppressed miR-27b expression (*** *p* < 0.0001) (**A**). MiR-27b inhibitor induced a downregulation in expression ((**A**); *** *p* < 0.0001) PINK1 gene expression ((**B**); *** *p* < 0.0001) PINK1 protein expression ((**C**); ** *p* < 0.005) were upregulated following FB_2_ exposure. FB_2_ increases p62 protein expression in Hek293 cells ((**D**); *** *p* < 0.0001).

Since Nrf2 transcriptionally regulates mitophagy, the effects of FB_2_ on proteins and genes involved in the process were analysed. Post-transcriptional regulation of mitophagy proteins was analysed by measuring miR-27b expression. MiR-27b expression was suppressed following exposure to FB_2_ (*p* < 0.0001). The miR-27b mimic showed no significant changes in miR-27b expression compared to the control, whereas the miR-27b inhibitor showed a significant decrease in miR-27b expression (*p* < 0.0001) (Figure 6A). FB_2_ increased *PINK1* transcript (*p* < 0.0001) (Figure 6B) and protein (*p* = 0.0014) (Figure 6C) expression levels in Hek293 cells. FB_2_ also increased expression of p62 (Figure 6D) (*p* < 0.0001). 

## 3. Discussion

The kidney is a primary target for fumonisin toxicity due to the accumulation and excretion of toxins via this organ [32,33]. However, limited biochemical studies exist demonstrating the effects of fumonisins in the human kidney_._ Unlike FB_2_, FB_1_ (a structural analogue) has well-established mechanisms, including induction of mitochondrial toxicity [10,12]. Apart from the canonical mechanism of sphingolipid metabolism disruption [16], little is known about FB_2_-induced toxicity_._ To date, the effects of FB_2_ on kidney cells and mitochondrial function have not been established. This study provides evidence that FB_2_ can induce mitophagy by preventing mitochondrial stress responses from occurring in kidney cells. This is the first study, to our knowledge, that illustrates mitochondrial toxicity induced by FB_2._ Furthermore, this study introduces a novel concept of FB_2_-induced post-transcriptional regulation (via miRNAs) of genes involved in mitophagy.

The MTT assay determined the cytotoxic potential of FB_2_; FB_2_ decreased cell viability in Hek293 cells (Figure 1) and induced cell death (Appendix A). This suggests that FB_2_ decreased NADH availability in cells; this altered the NAD^+^ ratio in cells, further compromising cell function and metabolism [34]. More importantly, studies have shown that a decline in the availability of NAD^+^ compromises mitochondrial function [35,36]. Therefore, the decrease in the NADH/NAD^+^ ratio promotes mitochondrial dysfunction.

A common consequence of mitochondrial dysfunction is depolarisation of the mitochondrial membrane [10]. An increase in ROS production contributes significantly to both depolarisation and stress of the mitochondria (as the mitochondrial respiratory chain has various sites for ROS production) [37]. Fumonisins are known to induce toxicity through increased ROS production via electron transport chain (ETC) inhibition and depolarisation of the mitochondrial membrane [10,12]. 

We show that FB_2_ can increase ROS production and cause depolarisation of the mitochondrial membrane in Hek293 cells (Figure 2). Elevations in ROS production are commonly attributed to aberrations in the mitochondrial ETC. Due to similarity in structure, FB_2_, like other fumonisins [10,12], possibly disrupts the ETC, resulting in excessive ROS production and ultimately depolarisation of the mitochondrial membranes (Figure 2).

A crucial marker for compromised mitochondrial activity is reduced ATP synthesis and increased mitochondrial stress markers [38]. The significant reduction in ATP production (Figure 3A) occurred due to increased mitochondrial stress and disturbances in the mitochondrial respiratory chain (mitochondrial dysfunction) post FB_2_ exposure. Previous studies have shown a direct correlation in the upregulation of HSP60 expression in response to mitochondrial stress and ROS, making it a suitable biomarker for the phenomenon [22]. FB_2_ significantly increased HSP60 protein expression (Figure 3B). Additionally, the decrease in ATP promotes stress in the mitochondria, causing the upregulation of HSP60. 

Increased mitochondrial stress induces an increase in the expression of mitochondrial sirtuins [39]. SIRT3 expression has been shown to increase following the induction of mitochondrial stress significantly [40]. Inhibition of SIRT3 has led to mitotoxicity and cell death due to an inadequate stress response. Fluctuations in SIRT3 expression can be considered a suitable biomarker for mitochondrial stress [41]. FB_2_ significantly decreased both SIRT3 mRNA and protein expressions (Figure 4A,B). SIRT3 (a class of NAD^+^-dependent deacetylases) is native to the mitochondria. SIRT3 expression and activity are dependent on the NADH/NAD^+^ ratio in the cell [42]. A decrease in the NADH/NAD^+^ ratio causes an increase in nicotinamide, an inhibitor of sirtuin function [43]. 

LONP1 reduces the effects of stress via the degradation of oxidatively damaged and misfolded proteins [20,44]. SIRT3 post-translationally regulates LONP1 via deacetylation. This causes a decrease in LONP1 protein expression when SIRT3 is elevated [45,46]. FB_2_ did not alter LONP1 expression despite the downregulation of SIRT3 (Figure 4C) and upregulation of ROS (Figure 2A). LONP1 (an ATP-dependent protease) contains a highly conserved ATPase domain with an AAA^+^ module and a proteolytic domain with an N-terminal domain [20,44]. FB_2_ depleted cellular ATP levels and consequently may have inhibited LONP1 catalytic activity and the degradation of oxidised proteins (Figure 3A). These findings suggest that FB_2_ prevents the upregulation of critical mitochondrial stress proteins necessary for the amelioration of stress.

FB_2_ induced mitochondrial stress and aberrations in mitochondrial function. Next, the effects of FB_2_ on Hek293 cell mitophagy were investigated. Mitophagy is a quality control process that allows for the degradation of damaged mitochondria, thus promoting homeostasis. PINK1 and p62 are critical proteins involved in the mitophagy process [19,25]. PINK1 is activated on the depolarised mitochondrial membrane allowing for the recruitment of other mitophagy proteins such as p62 [24,25,47]. Several promoters and inhibitors regulate the expression of PINK1 and p62.

A positive regulator of both PINK1 and p62 is the transcription factor Nrf2 [26,27]. Furthermore, p62 has been shown to increase the expression of Nrf2 [28]. Excessive ROS production activates phosphorylation pathways, triggering the phosphorylation of Nrf2 and promoting the translocation of pNrf2 to the nucleus, wherein PINK1 and p62 are transcribed [48]. The Nrf2–PINK1–p62 axis occurs as a means of cell survival [26,27]; however, excessive stimulation may result in cell death [49]. Figure 5 shows a significant increase in pNrf2, indicating mitophagy promotion. The finding is in agreement with excess ROS production (Figure 2A) and increased stress. 

Conversely, miRNAs have been implicated in the negative regulation of mitophagy. MiR-27b is a negative regulator of mitophagy as it can directly inhibit PINK1 expression via binding to the 3′-UTR of PINK1 mRNA [29]. FB_2_ significantly reduced miR-27b expression in Hek293 cells (Figure 6A). This coincided with the result obtained for the inhibitor of miR-27b, illustrating the toxins’ ability to act as an inhibitor for miRNAs expression (Figure 6A), thereby promoting mitophagy. Fumonisins have been shown to repress miR-27b expression [11]. However, FB_2_ induced inhibition of the miRNA and promoted mitophagy in Hek293 cells. 

FB_2_ induced a significant increase in the gene and protein expressions of PINK1 (Figure 6B,C). This was accompanied by correlating increases in p62 protein expression (Figure 6D). The data agree with data obtained for pNrf2 and miR-27b, suggesting that FB_2_ promotes mitophagy at a transcriptional and translational level. 

It can be deduced that the Nrf2–PINK1–p62 axis was positively regulated by FB_2_. The inhibition of miR-27b further supports that FB_2_ promotes mitophagy in Hek293 cells; this is the first study to our knowledge to report the promotion of mitophagy by a fumonisin in kidney cells.

The occurrence of mitophagy post FB_2_ exposure is in agreement with the suppressed mitochondrial stress responses, increased ROS, increased mitochondrial membrane depolarisation and compromised function of the organelle. However, mitophagy did not act as a cell survival mechanism as a significant reduction in cell viability was observed due to overstimulation of mitophagy, resulting in kidney cell death. 

This study provides insight into the role of FB_2_-induced mitotoxicity in Hek293 cells. FB_2_ increased ROS production, which increased mitochondrial stress and mitochondrial membrane depolarisation, dampened SIRT3 and LONP1 mitochondrial stress responses and promoted mitophagy. Furthermore, this study provides evidence of post-transcriptional regulation of PINK1 by miR-27b. 

## 4. Future Recommendations and Limitations

The present study was performed to establish the toxicity of FB_2_ at the estimated IC_50_ as per other numerous fumonisin toxicology studies. In doing so, preliminary data surrounding toxicity were provided. However, future studies need to include varied concentrations of FB_2_ and time periods to better understand FB2-induced toxicity. Furthermore, more cell lines that FB2 targets should be incorporated into studies. Additionally, future research can include comparative work between FB_1_ and FB_2_.

## 5. Materials and Methods

### 5.1. Materials

FB_2_ (F3771) was purchased from Sigma-Aldrich (St. Louis, MO, USA). Hek293 cells were obtained from American Type Culture Collection (Johannesburg, South Africa). Cell culture media and supplements were purchased from Lonza (Basel, Switzerland). Luminometry kits were purchased from Promega (Madison, WI, USA). Western blot reagents were purchased from Bio-Rad (Hercules, CA, USA). All other reagents were purchased from Merck (Darmstadt, Germany) unless otherwise stated.

### 5.2. Cell Culture and Treatment

Hek293 cells were cultured in 25 cm^3^ cell culture flasks using Dulbecco’s minimum essentials medium (DMEM) supplemented with 2.5 mM HEPES, 10% foetal bovine serum, 1% pen-strep-fungizone and 1% L-glutamine, maintained in a humidified incubator (37 °C, 5% CO_2_) until approximately 80% confluent. 

A stock solution of 20 mM FB_2_ was prepared in 0.1 M phosphate-buffered saline (PBS) and diluted using DMEM to achieve the concentrations for the MTT assay (0–500 µmol/L) and thereafter, for further experiments. All assays were performed three independent times and in triplicate.

### 5.3. Methyl Thiazol Tetrazolium (MTT) Assay

The cytotoxicity of FB_2_ in Hek293 cells was determined using the MTT assay. Briefly, 20,000 cells/well were seeded and allowed to adhere overnight in a 96-well microtitre plate (37 °C, 5% CO_2_). Thereafter, cells were incubated for 24 h with varying concentrations (0–500 µmol/L) of FB_2_. Control wells contained DMEM only. Following incubation, treatments were removed, cells were washed using 0.1 M PBS and incubated with MTT salt (20 µL; 5 mg/mL in 0.1 M PBS) and DMEM (100 µL) for 4 h. The MTT salt solution was then removed, and 100 µL of dimethyl sulphoxide (DMSO) was aliquoted per well and incubated at 37 °C for 1 h. Optical density was measured using a spectrophotometer (Bio-Tek uQuant Universal Microplate Spectrophotometer, Winoosiki, VT, USA) at 570 nm and a reference wavelength of 690 nm. Results were expressed as log concentration versus percentage cell viability. 

The IC_50_ was determined and used as the treatment concentration for all subsequent experiments. Non-linear regression analysis was used to estimate the IC_50_ (GraphPad Prism v5.0). This was in accordance with other toxicology studies using fumonisins that only used the IC_50_ for further testing [11,12]. All controls remained untreated (DMEM only) without the addition of FB_2._

### 5.4. ATP Assay

ATP concentration was measured using the CellTiter-Glo^®^ Luminescent Cell Viability Assay (Promega, #G7570). Cells were treated in 6-well plates for 24 h (37 °C; 5% CO_2_). Following treatment, 20,000 cells/well in 0.1 M PBS were seeded in an opaque 96-well microtitre plate in triplicate. As per the manufacturer’s instructions, the CellTiter-Glo^®^ Reagent was reconstituted, and 25 µL of reagent was added to each well. Plates were incubated in the dark for 20 min at room temperature, and luminescence was measured using a Modulus™ Microplate Reader (Turner Biosystems, Sunnyvale, CA, USA). Results were expressed as relative light units.

### 5.5. 2′,7′-Dichlorodihydrofluorescein Diacetate (H_2_DCFDA) Assay

ROS concentration was quantified using the DCF assay. Cells were treated in 6-well plates at 80% confluency for 24 h (37 °C, 5% CO_2_). Thereafter, 50,000 cells were aliquoted in four separate micro-centrifuge tubes. A stock solution of 80 mM H_2_DCF-DA (Thermo-Fisher, Waltham, MA, USA) was diluted using PBS to produce a 5 µmol/L working solution; 100 µL of the working solution was added to each micro-centrifuge tube (37 °C; 30 min). Cells were washed with PBS and subsequently centrifuged (400× *g*; 10 min). PBS was removed, and the process was repeated. Cells were re-suspended in PBS (200 µL) and transferred to an opaque 96-well microtitre plate. Fluorescence was measured using a Modulus™ Microplate Reader (Turner Biosystems, Sunnyvale, CA, USA) with an excitation wavelength of 503 nm and an emission wavelength of 509 nm as per Arumugam et al., 2018 [12]. Results were expressed as relative fluorescent units (RFU) compared to the control.

### 5.6. Mitochondrial Membrane Depolarisation—JC1-Mitoscreen

The mitochondrial membrane potential (Δψm) was measured using the JC-1 stain [50]. Following treatment, HepG2 cells (50,000 cells per treatment) were incubated in 200 μL of 5 μg/mL JC-1 stain (BD Biosciences, San Jose, CA, USA) (20 min, 37 °C). The stain was removed via centrifugation (400× *g*, 10 min, 24 °C), and the cells were washed twice with JC-1 staining buffer. Cells were re-suspended in JC-1 staining buffer (400 μL) and seeded in an opaque 96-well microtiter plate in triplicate (100 μL/well). A blank, consisting of only JC-1 staining buffer, was plated in triplicate (100 μL/well). Fluorescence was quantified on a Modulus™ microplate reader (Turner Biosystems, Sunnyvale, CA, USA). JC-1 monomers were measured with a blue filter (λex = 488 nm, λem = 529 nm) and JC-1 aggregates were measured with a green filter (λex = 524 nm, λem = 594 nm) Arumugam et al., 2018 [12]. The Δψm of the HEK293 cells is expressed as the fluorescence intensity ratio of membrane depolarisation and membrane polarization.

### 5.7. Western Blot

Following treatment for 24 h, cells were incubated with 150 µL Cytobuster™ Reagent (Novagen, San Diego, CA, USA, catalogue no. 71009) on ice for 30 min. Cells were mechanically lysed, transferred to 1.5 mL micro-centrifuge tubes, and centrifuged (400× *g*, 10 min, 4 °C). The supernatant containing crude protein isolates were aspirated and quantified. The bicinchoninic acid assay was used for protein quantification, and samples were standardised to a concentration of 1.5 mg/mL. Protein samples were boiled (5 min, 100 °C) in Laemmli Buffer (distilled water, glycerol, 10% SDS, β-mercaptoethanol, 0.5 M Tris-HCl (pH 6.8), 1% bromophenol blue and glycerol).

A Bio-Rad compact supply was used to electrophorese 25 µL samples (1 h, 150 V) in sodium dodecyl sulphate (SDS) polyacrylamide gels (4% stacking, 10% resolving). The Bio-Rad Trans-Blot^®^ Turbo Transfer system was used to transfer the separated proteins onto nitrocellulose membranes. All membranes were blocked in 5% Bovine Serum Albumin (BSA) in Tween 20-Tris buffer saline (TTBS: 150 mM NaCl, 3 mM KCl, 25 mM Tris, 0.05% Tween 20, dH_2_O, pH 7.5) for 1 h at RT. 

Membranes were then immuno-probed with the respective primary antibody (1:1000 dilution in 5% BSA) against phosphorylated (Ser40) Nrf2 (ab76026, Abcam, Cambridge, UK), SIRT3 (ab86671, Abcam), LONP1 (HPA002192, Sigma-Aldrich), PINK1 (a23707, Abcam), p62 (a56416, Abcam) and HSP60 (SAB4501464, Sigma-Aldrich) for 1 h at RT and overnight at 4 °C. Following incubation, membranes were washed five times for 10 min using 5 mL TTBS. Membranes were then incubated in HRP-conjugated secondary antibodies (Cell signalling Technology; anti-mouse (#7076P2), anti-rabbit (#7074S) 1:5000 in 5% BSA) for 1 h at RT. Membranes were then washed five times (10 min) using 5 mL TTBS and rinsed with distilled water. Clarity Western ECL Substrate detection reagent (400 µL) (Bio-Rad, Hercules, CA, US)) was added to membranes to detect protein bands, and images were captured using the Bio-Rad ChemiDoc™ XRS+ Imaging System. 

Membranes were quenched using 5% hydrogen peroxide for 30 min at 37 °C, blocked using 5% BSA and incubated in HRP-conjugated antibody for β-actin (A3854, Sigma-Aldrich) as a house-keeping protein. Results were analysed using Image Lab™ Software v6.0 (Bio-Rad, Hercules, CA, USA). Results were presented as relative band density of protein of interest divided by relative band density of the respective β-actin.

### 5.8. Quantitative PCR

#### 5.8.1. Treatment with the miR-27b Mimic and Inhibitor

Hek293 cells were grown in 25 cm^3^ flasks until 80% confluent. In a microcentrifuge tube, 15 µL of miR-27b mimic (hsa-miR-27b-3p; MSY0000419, Qiagen, Hilden, Germany), 72 µL serum-free DMEM and 3 µL of attractene were combined and mixed well. The process was repeated using 15 µL of miR-27b inhibitor (hsa-miR-27b-3p; MIN0000419, Qiagen). The micro-centrifuge tubes were incubated at RT to allow for complex formation. Each complex was then added to 2910 µL of DMEM in a dropwise manner. This was added to washed cells, and flasks were incubated for 24 h (37 °C; 5% CO_2_).

#### 5.8.2. RNA Isolation and Quantification

Following treatment for 24 h, cells were incubated with 500 µL Trizol and 500 µL PBS (5 min, RT). Samples were mechanically lysed, transferred to 2 mL micro-centrifuge tubes and stored (24 h, −80 °C). The samples were thawed at RT, followed by the addition of 100 µL chloroform and centrifugation (12,000× *g*, 10 min, 4 °C). The supernatant was transferred to fresh 2 mL micro-centrifuge tubes, and 250 µL isopropanol was added, followed by overnight storage at −80 °C. Thawed samples were then centrifuged (12,000× *g*, 20 min, 4 °C). The supernatant was discarded, and the pellet was washed in 500 µL cold ethanol (75%). Samples were centrifuged (7400× *g*, 15 min, 4 °C). The RNA pellets were air-dried (30 min, 24 °C) and re-suspended in 15 µL nuclease-free water. Quantification of the crude RNA was carried out using the Nanodrop2000 spectrophotometer (Thermo-Fisher Scientific, Waltham, MA, USA). RNA quality was determined using the A260/A280 ratio. All RNA samples were standardised to 500 ng/µL.

#### 5.8.3. Quantification of mRNA Expression

The cDNA was synthesised from the crude RNA samples using the iScript™ cDNA Synthesis kit (Bio-Rad, 107-8890, Hercules, CA, USA). 

Transcript levels of *SIRT3* and *PINK1* (Table 1) were assessed using the SsoAdvanced™ Universal SYBR^®^ Green Supermix (Bio-Rad, 1725270) and the CFX96 Touch™ Real-Time PCR Detection System (Bio-Rad, Hercules, CA, USA). The thermo-cycler conditions for each gene were as follows: initial denaturation (8 min, 95 °C), followed by 40 cycles of denaturation (15 s, 95 °C), annealing (40 s, Table 1), and extension (30 s, 72 °C). Data were normalised against the housekeeping gene, *GAPDH.* Results were analysed using the Livak and Schmittgen (2001) method and represented as fold change relative to the untreated control (2^−ΔΔCT^) [51]. 

#### 5.8.4. Quantification of miR-27b Expression

As per the manufacturer’s instructions, crude RNA was reverse transcribed into cDNA using the miScript II RT kit (Qiagen, 218161, Hilden, Germany)). The expression of miR-27b was assessed using the miScript SYBR Green PCR Kit (Qiagen, 218073, Hilden, Germany)) and CFX96 Touch™ Real-Time PCR Detection System (Bio-Rad, Hercules, CA, USA). The thermo-cycler conditions were as follows: initial denaturation (15 min, 95 °C), followed by 40 cycles of denaturation (15 s, 94 °C), annealing (30 s; 55 °C) and extension (30 s; 70 °C). Data were normalised against the housekeeping gene, RNU6 (218300, MS00033740). Results were analysed using the Livak and Schmittgen (2001) method and represented as fold change relative to the untreated control (2^−ΔΔCT^) [51].

### 5.9. Statistical Analysis

GraphPad Prism version 5.0 (GraphPad Prism Software Inc.) was used to perform all statistical analyses. Data were analysed using an unpaired t-test (data with 2 groups) and one-way analysis of variance (ANOVA) followed by a Bonferroni test for multiple group comparison. Data were considered significant at *p* < 0.05. 

## Figures and Tables

**Table 1:** Primer sequences with respective annealing temperatures for genes assessed.

Gene		Sequence (5′-3′)	Annealing Temperature (°C)
*SIRT3*	Sense	GAGCGGCCTCTACAGCAAC	60
	Anti-sense	GAGTAGTGAGTGACATTGGG	
*PINK1*	Sense	AAGCGAGGCTTTCCCCTAC	56
	Anti-sense	GCACTACATTGACCACCGATTT	
*GAPDH*	Sense	TCCACCACCCTGTTGCTGTA	-
	Anti-sense	ACCACAGTCCATGCCATCAC	

**Figure A1:**
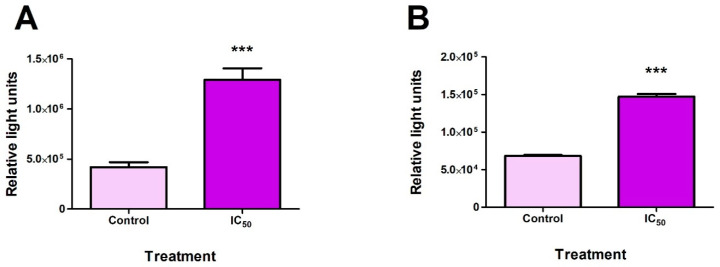
FB_2_-induced apoptosis in Hek293 cells. FB2 significantly increased the activity of caspase 9 ((**A**); *** *p* < 0.0001). A significant increase in executioner caspases 3/7 were observed following FB2 exposure ((**B**); *** *p* < 0.0001).

## Data Availability

Not applicable.

## Abbreviations

3′-untranslated region	3′-UTR
Dulbecco’s minimum essentials medium	DMEM
Electron transport chain	ETC
Fumonisin B_1_	FB_1_
Fumonisin B_2_	FB_2_
Heat shock protein 60	HSP60
Human embryonic kidney cells	Hek293
Lon protease 1	LONP1
Messenger RNA	mRNA
Methylthiazol tetrazolium	MTT
Micro-RNA 27b	miR-27b
Micro-RNA	miRNA
Nuclear factor (erythroid-derived 2)-like 2	Nrf2
Phosphate buffered saline	PBS
Phosphorylated Nrf2 (Ser40)	Nrf2
PTEN-induced putative kinase 1	PINK1
Quantitative PCR	qPCR
Reactive oxygen species	ROS
Sirtuin 3	SIRT3
The half-maximal inhibitory concentration	IC_50_
Ubiquitin-binding adaptor p62	p62

## Appendix A

FB2 induced apoptosis via the mitochondrial apoptotic pathway in Hek293 cells.

We measured the activity of initiator (caspase 9) and executioner caspases (caspase 3/7) of the mitochondrial apoptotic pathway using luminometry. FB2 significantly increased the activity of caspase 9 (*p* = 0.0003) (Figure A1A) and caspase 3/7 (*p* < 0.0001) (Figure A1B), suggesting the occurrence of cell death via the mitochondrial intrinsic apoptotic pathway.
